# Unveiling the Clinical and Biochemical Impact of Vitamin D Deficiency in Indian Rheumatoid Arthritis Patients: A Cross-Sectional Analysis

**DOI:** 10.7759/cureus.110517

**Published:** 2026-06-09

**Authors:** Shalini AS, Jayashankar CA, Venkataramana Kandi, Venkata Bharat Kumar Pinnelli, Surendra Babu T, Koshy T Sam, Manish GR, Snigdha Reddy, Akshay AS

**Affiliations:** 1 General Medicine, Vydehi Institute of Medical Sciences and Research Centre, Bengaluru, IND; 2 Internal Medicine, Vydehi Institute of Medical Sciences and Research Centre, Bengaluru, IND; 3 Clinical Microbiology, Prathima Institute of Medical Sciences, Karimnagar, IND; 4 Biochemistry, Vydehi Institute of Medical Sciences and Research Centre, Bengaluru, IND; 5 Anatomy, Vydehi Institute of Medical Sciences and Research Centre, Bengaluru, IND

**Keywords:** 25(oh)d, disease activity score-28, rheumatoid arthritis, serum 25-hydroxyvitamin d, vitamin d deficiency

## Abstract

Background

Rheumatoid arthritis (RA) is a chronic autoimmune disease characterized by persistent synovial inflammation, joint destruction, and systemic complications. Despite therapeutic advances, disease activity remains variable, and modifiable factors influencing severity are under investigation. Vitamin D, beyond its role in bone metabolism, exerts immunomodulatory effects and may influence RA pathogenesis. However, evidence regarding its association with disease activity remains inconsistent. This study aimed to evaluate the clinical and biochemical correlates of serum 25-hydroxyvitamin D (25(OH)D) levels in RA patients within an Indian cohort.

Methodology

A cross‑sectional study was conducted among 50 newly diagnosed, treatment‑naïve patients with RA, aged over 18 years, at a tertiary care center in India. Demographic details, tender and swollen joint counts, visual analog scale (VAS) scores, erythrocyte sedimentation rate (ESR), C‑reactive protein (CRP), and Disease Activity Score 28 (DAS‑28) were recorded, while serum 25(OH)D concentrations were measured and categorized as normal, insufficient, or deficient. Statistical analyses were performed using SPSS version 20, with descriptive statistics expressed as mean ± SD and interquartile ranges (IQRs). Group differences in serum 25(OH)D were assessed using one‑way analysis of variance, followed by Tukey’s post hoc test. To determine the structural independent effects of circulating vitamin D levels on baseline RA severity, a multiple linear regression analysis was performed on the cohort. Receiver operating characteristic (ROC) curve analysis was conducted to determine sensitivity, specificity, positive predictive value (PPV), negative predictive value (NPV), and area under the curve (AUC) for ESR and DAS‑28 in predicting disease activity, with statistical significance set at p-values ≤0.05.

Results

Of the 50 patients tested, rheumatoid factor (RF) was positive in 46 (92.00%), while the remaining four patients were negative for RF but positive for anti‑cyclic citrullinated peptide (anti‑CCP) antibodies. The mean age was 40.32 ± 3.46 years (IQR = 35-46). The median tender joint count was 2.0 (IQR = 1-3), and the swollen joint count was 4.00 (IQR = 2.00-5.00). The mean DAS‑28 score was 4.11 ± 1.50 (IQR = 3.20-5.10), indicating predominantly moderate disease activity. Pain intensity assessed by VAS averaged 4.58 ± 1.88 (IQR = 3-6). ESR averaged 30.92 ± 18.89 mm/hour (IQR = 20-42), while CRP was 5.18 ± 4.65 mg/dL (IQR = 2.10-7.80). Serum 25(OH)D levels were relatively low, with a mean of 26.84 ± 3.96 ng/mL (IQR = 18.00-32.00), highlighting the prevalence of vitamin D deficiency in this population. The multiple linear regression model significantly predicted continuous DAS‑28 scores (F (3, 46) = 21.34, p < 0.001), explaining 58.2% of the variance (R² = 0.582). Serum 25(OH)D emerged as a highly significant and independent inverse determinant of baseline disease severity (B = −0.091, SE = 0.011, β = −0.741, p < 0.001). The area under the curve was 0.87 for ESR and 0.82 for serum 25(OH)D, underscoring good overall accuracy in predicting disease severity.

Conclusions

Vitamin D deficiency is a robust independent determinant of disease activity in Indian RA patients, underscoring the importance of monitoring vitamin D status alongside composite disease activity scores.

## Introduction

Rheumatoid arthritis (RA) is a chronic autoimmune disorder characterized by symmetrical polyarthritis, leading to progressive joint damage, disability, and reduced quality of life. The global prevalence of RA ranges between 0.5% and 1.0%. Autoimmune targeting of self‑antigens within synovium, cartilage, and bone underlies the majority of joint injury and functional impairment. Despite advances in disease‑modifying therapies, disease activity and progression remain highly variable among individuals [1].

Traditionally recognized for its role in calcium metabolism, vitamin D is now established as a key immunomodulatory hormone [2]. It exists in two principal forms, namely, vitamin D2 (ergocalciferol), derived mainly from dietary sources such as fungi and plants, and vitamin D3 (cholecalciferol), obtained from animal products or synthesized in the skin via ultraviolet B (UVB)-mediated conversion of 7‑dehydrocholesterol. Activation of vitamin D requires sequential hydroxylation. In the liver, the cytochrome P450 enzyme 25‑hydroxylase (CYP2R1) converts cholecalciferol to 25‑hydroxyvitamin D (25(OH)D). Subsequently, renal 1α‑hydroxylase (CYP27B1) catalyzes the conversion of 25(OH)D to the biologically active metabolite 1,25‑dihydroxyvitamin D (1,25(OH)₂D) within the proximal convoluted tubules. Active vitamin D then exerts feedback inhibition on 1α‑hydroxylase and induces 24‑hydroxylase activity, which degrades 25(OH)D to the inactive metabolite 24,25‑dihydroxyvitamin D [3]. Immune cells, including macrophages, dendritic cells, B lymphocytes, and thymus (T) lymphocytes, express vitamin D receptors. Vitamin D modulates immune responses by suppressing T-helper type 1 (Th1) and Th17 activity, reducing pro‑inflammatory cytokine secretion, and inhibiting autoantibody production [3,4]. This suggests a protective role of vitamin D activities in alleviating inflammation, potentially associated with autoimmune and infectious conditions.

Serum 25(OH)D is considered the best metabolite to measure when assessing vitamin D status and homeostasis because it is the major circulating form, reflecting both dietary intake and endogenous synthesis. Unlike the active metabolite 1,25(OH)₂D, which has a short half‑life and is tightly regulated by parathyroid hormone and calcium levels, 25(OH)D has a longer half‑life and provides a more stable indicator of overall vitamin D reserves. Therefore, measuring 25(OH)D captures the body’s vitamin D supply available for conversion into the active hormone, making it the most reliable marker for evaluating deficiency, sufficiency, and its clinical impact on conditions such as RA. Several studies have reported lower serum vitamin D levels in patients with RA compared to healthy controls, although evidence regarding its association with disease activity remains inconsistent [5-7]. Moreover, data specific to South Indian populations are limited. The present study aimed to systematically evaluate the association between serum 25(OH)D concentrations and baseline disease activity in newly diagnosed, treatment‑naïve Indian patients with RA. Specifically, the objective was to delineate the clinical and biochemical correlates of vitamin D status and to determine whether deficiency serves as an independent determinant of disease severity within this cohort.

## Materials and methods

This prospective, cross‑sectional observational study included 50 newly diagnosed RA patients who fulfilled the 2010 classification criteria of the European League Against Rheumatism (EULAR) and the American College of Rheumatology (ACR). The study was conducted between January 2020 and July 2021 at the Vydehi Institute of Medical Sciences and Research Centre, Bengaluru, India. The institutional ethics committee approved the study protocol (letter number: VIEC/2019/APP/101), and written informed consent was obtained from all participants before enrollment.

Inclusion and exclusion criteria

Patients aged ≥18 years, newly diagnosed with RA, and naïve to both vitamin D supplementation and disease‑modifying anti‑rheumatic drugs were recruited through purposive sampling. Exclusion criteria included individuals with liver disease, malabsorption syndromes, chronic kidney disease, hyperparathyroidism, thyroid disorders, diabetes mellitus, other systemic illnesses, autoimmune conditions such as systemic lupus erythematosus, or endocrine abnormalities that could influence serum 25(OH)D activity. Patients receiving enzyme‑inducing drugs, calcium supplementation, or vitamin D supplementation within the preceding six months were also excluded.

Sample size estimation

The sample size was calculated as n = [(Z_α_+Z_β_)/C]^2^+3, yielding a value of 50. In this computation, Z_α_ represented the standard normal deviate at a 99% confidence interval (2.5758), and Z_β_ corresponded to the standard normal deviate at 90% power (1.2816). The constant C was derived as 0.56 using Fisher’s Z transformation formula: C = 0.5⋅ln⁡(1+r/1−r), where r denoted the expected correlation coefficient (0.51) [8].

Procedure

Only patients fulfilling the 2010 ACR-EULAR classification criteria for RA were included [9]. A detailed general physical examination was conducted and documented in the case record proforma, encompassing vital signs, systemic evaluation, age at symptom onset, clinical course since diagnosis, distribution and number of joints involved, associated manifestations such as swelling and pain, and relevant drug history.

The study was conducted in Bengaluru, Karnataka (12.97°N latitude). The region experiences a tropical savanna climate with ~2,800-3,000 hours of sunshine annually, providing ample UVB exposure. Despite this, urban lifestyle patterns, indoor occupations, cultural clothing practices, and air pollution limit effective sun exposure among patients.

Rheumatoid factor (RF) and anti‑cyclic citrullinated peptide (anti‑CCP) antibodies were quantified using chemiluminescence immunoassay (CLIA). Serum 25(OH)D concentrations were determined by an enhanced CLIA on a VITROS® 5600 automated analyzer using the manufacturer’s proprietary microwell kit (Quidel Ortho, USA). All blood samples were collected at baseline during the cross‑sectional recruitment period, thereby permitting correlation only with contemporaneous disease activity and inflammatory markers. Serum 25(OH)D status was subsequently categorized as normal (30-70 ng/mL; 75 nmol/L), insufficient (20.0-29.9 ng/mL; 52-72 nmol/L), and deficient (<20 ng/mL; 50 nmol/L). Erythrocyte sedimentation rate (ESR) was estimated using standard hematological techniques.

Disease activity was assessed using the Disease Activity Score in 28 joints (DAS‑28), as recommended by the ACR. The DAS‑28 was calculated using the following formula: DAS−28=0.56√28TJC+0.28√28SJC+0.70⋅ln⁡(ESR)+0.014⋅VAS

where TJC is the tender joint count, SJC is the swollen joint count, ESR is the erythrocyte sedimentation rate, and VAS is the visual analog scale. Disease activity was classified as remission (<2.6), low (2.6-3.2), moderate (3.2-<5.1), and high (>5.1).

Statistical analysis

Statistical analyses were conducted using SPSS Statistics for Windows, Version 20.0 (IBM Corp., Armonk, NY, USA) and Microsoft Excel 2019 (Microsoft Corp., Redmond, WA, USA). The distribution of continuous variables was assessed for normality using the Shapiro-Wilk test. Normally distributed data were expressed as mean ± standard deviation (SD), whereas non‑normally distributed or ordinal data were summarized as median with interquartile range (IQR). Categorical variables were presented as absolute frequencies and percentages (n%). In our study, there were no missing data; only samples with complete clinical and biochemical information were included in the analysis. Therefore, no imputation or special handling of missing values was required. To examine the distribution of clinical joint counts across predefined severity categories (e.g., tender and swollen joint groups), a chi‑square (χ²) goodness‑of‑fit test was applied to determine whether observed frequencies differed significantly from an equal distribution. Differences in continuous clinical and biochemical parameters, particularly serum 25(OH)D levels, across disease activity stages (remission, low, moderate, and high activity, defined by DAS‑28) were analyzed using one‑way analysis of variance (ANOVA). A separate one‑way ANOVA was performed to compare biochemical markers across three vitamin D status groups (normal, insufficient, and deficient). Assumptions for ANOVA (normality and homogeneity of variances) and linear regression (linearity, normal distribution of residuals, and homoscedasticity) were formally assessed and met before conducting analyses. For all significant omnibus ANOVA results, Tukey’s honestly significant difference (HSD) post hoc test was employed for pairwise comparisons. To determine the structural independent effects of circulating vitamin D levels on baseline RA severity, a multiple linear regression analysis was performed on the cohort. The diagnostic performance of inflammatory markers in predicting high disease activity was assessed using receiver operating characteristic (ROC) curve analysis. Discriminatory accuracy was quantified by the area under the curve (AUC), and diagnostic thresholds were validated using sensitivity, specificity, positive predictive value (PPV), and negative predictive value (NPV). All statistical tests were two‑tailed, and a p‑value <0.05 was considered statistically significant.

## Results

A total of 50 newly diagnosed, treatment‑naïve patients with RA were enrolled in this prospective cross‑sectional study. The cohort demonstrated a female predominance, with 31 (62%) women and 19 (38%) men, yielding a female‑to‑male ratio of 1.6:1. The overall mean age was 40.32 ± 3.46 years (IQR = 35-46), spanning 19 to 68 years. None of the participants had documented systemic comorbidities or endocrine abnormalities at baseline. Clinically, the cohort was characterized by moderate systemic disease activity, reflected in a mean DAS‑28 score of 4.11 ± 1.50 (IQR = 3.2-5.1). Consistent with their treatment‑naïve status, systemic inflammation was evident, with elevated mean ESR (30.92 ± 18.89 mm/hour; IQR = 20-42) and CRP (5.18 ± 4.65 mg/dL; IQR = 2.1-7.8). A widespread micronutrient deficit was also noted, with mean serum 25(OH)D levels of 26.84 ± 3.96 ng/mL (IQR = 18-32). For statistical accuracy, parametric values were reported for normally distributed variables, while skewed joint counts were expressed non‑parametrically, yielding a median tender joint count of 2 (IQR = 1-3) and a median swollen joint count of 4 (IQR = 2-5). Serological evaluation revealed a high prevalence of autoimmune markers: RF was positive in 46 (92%) patients, while the remaining 4 (8%) were RF‑negative but anti‑CCP antibody‑positive. The complete sociodemographic, clinical, biochemical, and serological characteristics of the study population are summarized in Table 1.

**Table 1:** Baseline demographic, clinical, biochemical, and serological profile of the study cohort (N = 50). SD: standard deviation; anti-CCP: anti-cyclic citrullinated peptide; IQR: interquartile range; DAS-28: Disease Activity Score 28; VAS: visual analog scale; ESR: erythrocyte sedimentation rate; CRP: C-reactive protein; serum 25(OH)D: serum 25-hydroxyvitamin D

Variable	Value/Prevalence	Value
Age (years)	Mean ± SD (range)	40.32 ± 3.46 (19–68)
	IQR	35–46
Sex	Female, n (%)	32 (64.0%)
	Male, n (%)	18 (36.0%)
Comorbidities	Nil, n (%)	50 (100.0%)
Serological status	Rheumatoid factor positive, n (%)	46 (92.0%)
	Anti-CCP antibodies positive, n (%)	4 (8.0%)
Tender joints	Median (IQR)	2 (1–3)
Swollen joints	Median (IQR)	4 (2–5)
DAS-28	Mean ± SD	4.11 ± 1.50
	IQR	3.2–5.1
VAS (0–10 cm)	Mean ± SD	4.58 ± 1.88
	IQR	3–6
ESR (mm/hour)	Mean ± SD	30.92 ± 18.89
	IQR	20–42
CRP (mg/dL)	Mean ± SD	5.18 ± 4.65
	IQR	2.1–7.8
Serum 25(OH)D (ng/mL)	Mean ± SD	26.84 ± 3.96
	IQR	18–32

The majority of patients were between 30 and 60 years of age. Overall, nine (18%) participants were between 18 and 25 years of age, and only three (6%) were older than 60 years. The largest subgroup was in the 51-55‑year age range, comprising 10 (20%) patients. The age distribution of the study population is summarized in Table 2.

**Table 2:** Age-wise distribution among the study participants (N = 50).

Age (years)	n (%)
19–25	9 (18)
26–30	5 (10)
31–35	8 (16)
36–40	4 (8)
41–50	7 (14)
51–55	10 (20)
56–60	4 (8)
>60	3 (6)
Total	50 (100)

Chi‑square goodness‑of‑fit analyses were applied to the tender and swollen joint count categories to examine whether the observed distributions deviated significantly from a uniform expectation. This approach was intended as an exploratory assessment of distributional imbalance across joint categories rather than to test a clinically driven hypothesis. The results are presented to provide a statistical description of how joint involvement was distributed within the cohort. During the study period, 21 (42%) patients presented without any tender joints (p > 0.01). A total of 22 (44%) patients demonstrated involvement of 1-5 tender joints, which was statistically significant (p < 0.01). Five (10%) patients exhibited tenderness in 6-10 joints (p < 0.01), while only two (4%) patients had more than 10 tender joints affected (p < 0.01). These findings highlight that the majority of patients had either no tender joints or limited involvement (≤5 joints), whereas extensive joint tenderness was relatively uncommon. The detailed distribution of tender joint counts is presented in Table 3.

**Table 3:** Distribution of tender joints among the study participants (N = 50). Data are expressed as absolute frequencies and percentages (n%). A chi-square goodness-of-fit test was applied to analyze the categorical distribution of tender joint counts across predefined severity categories to evaluate deviations from an equal distribution. A p-value <0.05 is considered statistically significant.

Number of tender joints	n (%)	Chi-square value	P-value
Nil	21 (42%)	41.36	<0.01
1–5	22 (44%)		
6–10	5 (10%)		
>10	2 (4%)		
Total	50 (100%)		

Examination of swollen joint involvement demonstrated variable patterns across the study cohort. Overall, 19 (38%) patients presented without any swollen joints. The largest subgroup comprised 20 (40%) patients who had involvement of 1-5 swollen joints, which was statistically significant (p < 0.01). Seven (14%) patients exhibited swelling in 6-10 joints (p < 0.01), while four (8%) patients had more than 10 swollen joints affected (p < 0.01). These findings indicate that the majority of patients had either no swelling or limited joint involvement, whereas extensive swelling affecting more than 10 joints was relatively uncommon. The detailed distribution of swollen joint counts is summarized in Table 4.

**Table 4:** Distribution of swollen joints among the study participants (N = 50). Data are expressed as absolute frequencies and percentages (n%). A chi-square goodness-of-fit test was utilized to determine whether the observed concentration of patients across swollen joint categories differed significantly from a uniform distribution. A p-value <0.05 is considered statistically significant.

Number of swollen joints	n (%)	Chi-square value	P-value
Nil	19 (38%)	33.20	<0.01
1–5	20 (40%)		
6–10	7 (14%)		
>10	4 (8%)		
Total	50 (100%)		

A one-way ANOVA demonstrated significant differences in serum 25(OH)D levels across the four DAS-28 disease activity categories. Patients in the remission group (n = 11, 22%) exhibited the highest mean vitamin D levels (32.40 ± 14.80 ng/mL). Subsequent Tukey’s post hoc pairwise comparisons revealed that serum 25(OH)D levels dropped progressively and significantly as disease activity increased. Relative to the remission reference cohort, significantly lower mean vitamin D concentrations were observed in both the low disease activity group (26.20 ± 6.60 ng/mL, p < 0.01) and the moderate disease activity group (18.50 ± 5.20 ng/mL, p < 0.01). The lowest serum concentrations were recorded among patients with high disease activity (12.10 ± 3.20 ng/mL), representing a highly significant deficit relative to the remission group (p < 0.001). Taken together, these data establish a distinct, inverse relationship between circulating vitamin D status and clinical disease severity (Table 5).

**Table 5:** Comparison of serum 25(OH)D levels across different stages of disease activity in patients (N = 50). Serum 25(OH)D status was categorized as: normal (30-70 ng/mL), insufficient (20.0-29.9 ng/mL), and deficient (<20 ng/mL). Group means were evaluated and compared using ANOVA. Pairwise post-hoc comparisons against the reference cohort (remission group) were performed utilizing Tukey’s HSD test. The omnibus F-statistic reflects the overall variation between the groups. Statistical significance for the tests was established at p < 0.01. A p-value <0.05 is considered statistically significant. ANOVA: analysis of variance; serum 25(OH)D: serum 25-hyroxyvitamin D; DAS-28: Disease Activity Score 28; SD: standard deviation; HSD: honestly significant difference

Disease activity (DAS-28)	n (%)	Serum 25(OH)D, mean ± SD (ng/mL)	Test statistic (F-value)	ANOVA p-value	Tukey’s HSD (vs. remission)	Clinical and statistical interpretation
Remission (<2.6)	11 (22%)	32.40 ± 14.80	21.34	<0.01	Baseline group	Patients in remission have the optimal/highest vitamin D levels
Low (2.6–3.2)	8 (16%)	26.20 ± 6.60		<0.01	Significant reduction	Significant drop: vitamin D is significantly lower than the remission group
Moderate (3.2–<5.1)	17 (34%)	18.50 ± 5.20		<0.01	Significant reduction	Marked deficiency: vitamin D drops into deficient levels as disease activity rises
High (>5.1)	14 (28%)	12.10 ± 3.20		<0.001	Highly significant reduction	Severe deficiency: highest disease activity correlates directly with the lowest vitamin D levels

The multiple linear regression model significantly predicted continuous DAS‑28 scores (F (3, 46) = 21.34, p < 0.001), explaining 58.2% of the variance (R² = 0.582). Serum 25(OH)D emerged as a highly significant and independent inverse determinant of baseline disease severity (B = −0.091, SE = 0.011, β = −0.741, p < 0.001). This indicates that for every unit increase in vitamin D concentration, the composite disease score decreased by 0.091 points after adjustment for demographic covariates. In contrast, patient age (B = 0.004, p = 0.662) and biological sex (B = 0.112, p = 0.648) did not contribute significantly to the variance in disease activity within this cohort (Table 6).

**Table 6:** Multiple linear regression analysis predicting baseline DAS-28 scores (N = 50). Data were evaluated using a simultaneous-entry multiple linear regression model with the continuous composite DAS-28 score serving as the dependent variable. Biological sex was dummy-coded (female = 1, male = 0). The overall model significance was validated via the omnibus F-test, and individual predictor variables were evaluated using unpaired Student’s t-tests. Model variance explanation was quantified using R² = 0.582. A p-value <0.05 is considered statistically significant. SE: standard error; DAS: Disease Activity Score 28; serum 25(OH)D: serum 25-hydroxyvitamin D

Variable	Unstandardized coefficient (B)	SE	Standardized coefficient (β)	t-statistic	P-value	95% confidence interval
Intercept (model fit: F = 21.34)	6.521	0.482	—	13.53	<0.001	5.551 to 7.491
Serum 25(OH)D (ng/mL)	-0.091	0.011	-0.741	-8.27	<0.001	-0.113 to -0.069
Age (years)	0.004	0.009	0.038	0.44	0.662	-0.014 to 0.022
Sex (female vs. male)	0.112	0.243	0.039	0.46	0.648	-0.377 to 0.601

ROC curve methodology was employed to evaluate the discriminative ability of selected biochemical parameters in relation to RA disease activity. Although ESR is a component of the DAS-28 score, the ROC analysis was used to illustrate the extent to which ESR and other markers individually distinguished between disease activity states in this population. This was intended as a consistency check of the internal validity of DAS-28 and to highlight the relative performance of biochemical variables. Both ESR and serum 25(OH)D demonstrated strong discriminatory capacity, with specificity exceeding 95% and sensitivity reaching 88%. PPVs were high at 86% for ESR and 78% for serum 25(OH)D, whereas NPVs were comparatively modest at 42% and 45%, respectively. The AUC was 0.87 for ESR and 0.82 for serum 25(OH)D, confirming good overall accuracy in predicting disease activity. These results highlight ESR and serum 25(OH)D as robust markers for evaluating inflammatory burden and disease severity in RA. Detailed ROC parameters are presented in Table 7.

**Table 7:** Specificity, sensitivity, PPV, NPV, and accuracy of parameters (N = 50). PPV: positive predictive value; NPV: negative predictive value, AUC: area under the curve; serum 25(OH)D: serum 25-hydroxyvitamin D; ESR: erythrocyte sedimentation rate; CRP: C-reactive protein

Variable	Specificity (%)	Sensitivity (%)	PPV (%)	NPV (%)	AUC (%)
ESR	95	82	86	42	0.87 (87)
CRP	65	56	47	38	0.58 (58)
Serum 25(OH)D	88	84	78	45	0.82 (82)

## Discussion

RA is a chronic autoimmune disorder characterized by persistent synovial inflammation, progressive joint destruction, and periarticular bone involvement. It remains one of the most prevalent connective tissue diseases worldwide. Despite advances in disease‑modifying therapies, the precise etiology and pathogenesis of RA are not fully understood, and disease activity varies considerably among individuals. Among the various factors influencing outcomes, vitamin D has emerged as a potential immunomodulatory agent, though its role in RA activity remains controversial. Several studies have reported conflicting findings, underscoring the need for further investigation [10,11].

Vitamin D is central to calcium and phosphorus homeostasis, regulating bone growth and mineralization through intestinal absorption and extracellular fluid saturation. Beyond skeletal health, vitamin D influences immune cell function, cytokine regulation, and inflammatory pathways, suggesting a plausible link to RA pathogenesis [10]. Recent trials and meta‑analyses have demonstrated that vitamin D supplementation may reduce DAS‑28, ESR, and CRP, while improving serum vitamin D status in RA patients [10,11]. However, heterogeneity persists, with some studies reporting limited effects on pain scores and functional outcomes, highlighting the complexity of vitamin D’s role in RA and the need for standardized supplementation protocols.

In the present study, RA was observed to be approximately three times more prevalent among females compared to males, with peak incidence in the 40-50‑year age group. The cohort comprised 31 (62%) females and 19 (38%) females, yielding a female‑to‑male ratio of 1.6:1. The mean age of female participants was 45.56 ± 1.22 years, while that of male participants was 39.89 ± 1.36 years. This predominance of female patients and comparable age distribution has been consistently reported in earlier studies [12,13]. A recent Global Burden of Disease (GBD) analysis demonstrated that, in India, the age‑standardized prevalence of RA was consistently greater in females, with peak incidence occurring between 65 and 69 years and peak prevalence between 75 and 79 years [14]. Our findings align with these national trends, though the mean age of onset in our cohort was younger, reflecting regional variation in disease presentation. Comparable female predominance has also been documented in multicenter Indian cohorts [15].

Participants in our study were aged over 18 years, with only three (6%) patients above 60 years and nine (18%) patients between 18 and 25 years. The mean age of the cohort was 40.32 ± 3.46 years (IQR = 35-46), with the highest proportion of cases (20%) observed in the 51-55‑year age group. These findings suggest a relatively younger age of presentation compared to several international cohorts. For instance, Kobak reported a mean age of 46.27 ± 11.87 years, with most patients between 35 and 57 years of age [13], while Oelzner et al. described a mean age of 54.7 years in a German cohort [16]. The GBD analysis similarly reported peak incidence in India at 65-69 years and peak prevalence at 75-79 years [14]. Compared to these national estimates, our cohort demonstrates an earlier age of onset, aligning with prior Indian prevalence studies that noted younger presentation in community‑based samples [15]. This discrepancy may reflect differences in referral patterns, regional demographics, or healthcare access.

Chi‑square goodness‑of‑fit analyses provided descriptive insight into joint involvement. The majority of patients (62%) had ≤5 tender joints and 68% had ≤5 swollen joints, while extensive involvement (>10 joints) was relatively uncommon (<15%). These findings highlight the heterogeneity of joint manifestations and emphasize that most patients presented with limited joint tenderness or swelling. Notably, the moderate DAS-28 average was heavily driven by the cohort’s high inflammatory markers (mean ESR = 30.92 mm/hour) and elevated subjective pain scores (mean VAS = 4.58), underscoring the combined influence of objective and patient‑reported measures in shaping disease activity.

In our study, the mean serum 25(OH)D concentrations were 32.40 ± 14.82 ng/mL in the normal group, 26.20 ± 6.61 ng/mL in the insufficient group, and 12.09 ± 3.16 ng/mL in the deficient group. The overall mean concentration (26.84 ± 3.96 ng/mL) indicates that the majority of patients fell within the insufficient category. Despite Bengaluru’s year-round availability of sunlight, several factors appear to limit effective cutaneous UVB exposure in this cohort. Restricted outdoor activity, clothing practices that reduce skin exposure, and urban air pollution collectively contribute to inadequate vitamin D synthesis. These findings explain the persistence of vitamin D deficiency even under seemingly favorable climatic conditions.

A significant correlation was observed between serum 25(OH)D levels and disease activity. Tukey’s post hoc analysis confirmed statistically significant differences between groups, particularly in patients with normal vitamin D status. These results demonstrate a clear inverse association between vitamin D status and RA severity. However, given the cross‑sectional design, causality cannot be inferred, and the relationship may be bidirectional. Active disease itself could contribute to reduced vitamin D levels through systemic inflammation, reduced mobility, or diminished sunlight exposure. Liu and Wen, in a study of 280 treatment‑naïve RA patients and 140 healthy controls, reported significantly lower mean serum 25(OH)D levels in RA patients (12.24 ± 6.68 ng/mL vs. 21.08 ± 7.14 ng/mL; p < 0.05), with vitamin D levels inversely associated with DAS‑28 scores (β = −0.164, p = 0.018) [17]. The concordance between our results and those of Liu and Wen highlights a consistent association between vitamin D status and RA disease activity, supporting the rationale for future research into its potential role as a biomarker and therapeutic target.

Emerging evidence also suggests that vitamin D deficiency may contribute to neuropathic complications in RA. A study reported mean serum 25(OH)D levels of 16.60 ± 2.7 ng/mL in RA patients with neuropathic pain compared to 29.80 ± 8.0 ng/mL in controls, with deficiency observed in 80% of affected individuals. Reduced vitamin D levels correlated inversely with DAS‑28 scores, pain intensity, and neuropathy prevalence [18]. These findings suggest that vitamin D status may not only influence inflammatory activity but also contribute to the burden of neuropathic manifestations, highlighting supplementation as a potential adjunctive therapeutic strategy.

Globally, vitamin D deficiency is widespread, affecting up to 37.3% of the population, and nearly one‑third of individuals in the United States present with serum 25(OH)D levels <20 ng/mL. Among RA patients, deficiency is even more prevalent, with multiple studies reporting mean concentrations between 15 and 30 ng/mL. These levels were consistently inversely correlated with DAS‑28, CRP, and ESR. Supplementation trials have yielded mixed results, with doses ranging from 4,000 IU daily to 300,000 IU single doses. While some studies reported significant improvements in pain and cytokine reduction, others found no effect, underscoring the need for standardized dosing strategies in RA management [19].

Our findings of a significant inverse correlation between serum 25(OH)D levels and DAS‑28 are consistent with Indian data. Chandrashekara and Patted studied 150 RA patients and reported that 49% had DAS28‑CRP >2.6 with serum 25(OH)D <20 ng/mL. Supplementation significantly reduced mean DAS28‑CRP from 3.68 ± 0.93 to 3.08 ± 1.11, indicating that correction of vitamin D deficiency contributed to short‑term improvement in disease activity [20]. Similarly, Bose et al. conducted a meta‑analysis of 15 Indian studies and reported mean serum 25(OH)D concentrations of 19.99 ng/mL (95% confidence interval = 16.49-24.23). Vitamin D deficiency (<20 ng/mL) was present in 56% of patients, while insufficiency (20-30 ng/mL) was noted in 20%. Importantly, a negative correlation was observed between serum 25(OH)D levels and DAS‑28, reinforcing the association between low vitamin D status and higher RA activity [21]. Comparable findings have been observed in psoriasis, where vitamin D deficiency was associated with increased disease severity [22,23]. This suggests that inadequate vitamin D status may lower the threshold for flares in autoimmune conditions, including RA, when triggering factors occur.

ROC curve analysis further evaluated the discriminatory performance of biochemical markers. Both ESR and serum 25(OH)D demonstrated strong discriminatory performance, with specificity exceeding 95% for ESR and 88% for serum 25(OH)D. PPVs were high (86% for ESR and 78% for serum 25(OH)D), confirming their reliability in identifying patients with active disease. However, NPVs were comparatively modest (42% and 45%, respectively), indicating that a “normal” result cannot reliably exclude significant inflammatory activity. The AUC was 0.87 for ESR and 0.82 for serum 25(OH)D, underscoring good overall accuracy in predicting disease severity. As ESR is already incorporated into DAS‑28, its strong ROC performance was expected and primarily served as a consistency check. The comparative utility of vitamin D alongside conventional inflammatory markers is noteworthy, but its role as an independent biomarker remains unproven.

Taken together, our findings, supported by both national and international evidence, reinforce the role of vitamin D deficiency as a modifiable risk factor for heightened disease activity in RA. Routine screening and correction of vitamin D deficiency may therefore represent a simple, cost‑effective adjunct to existing therapeutic strategies, with potential to improve disease outcomes and quality of life in RA patients.

Study limitations

This study has several limitations that warrant consideration. First, the cross‑sectional design restricts causal inference, as all measurements were obtained at a single baseline time point, permitting only contemporaneous associations between vitamin D status, disease activity, and biochemical parameters. Second, although multivariable regression was performed, the model adjusted only for age, sex, and serum vitamin D levels. Important potential confounders, including body mass index, smoking status, sunlight exposure, nutritional status, socioeconomic background, and physical activity, were not incorporated due to data constraints. Third, the sample size was modest and derived from a single center, which may limit generalizability to broader populations. Finally, laboratory assays were conducted at baseline only, without longitudinal follow‑up, precluding assessment of temporal trends or treatment effects. Future research should not only involve larger, multicentric cohorts with comprehensive adjustment for lifestyle and socioeconomic variables but also longitudinal designs that evaluate changes in serum vitamin D levels before and after achieving remission with RA treatment (in the absence of vitamin D supplementation) to better clarify the directionality of the observed association.

## Conclusions

This study establishes a clear and statistically significant association between serum vitamin D concentrations and RA disease activity. Patients with greater disease activity consistently demonstrated lower vitamin D levels, underscoring the relevance of vitamin D status in the clinical profile of RA. While the cross‑sectional design limits causal inference, the strength of the observed relationship highlights vitamin D as a factor that warrants systematic consideration in future research on RA pathophysiology and management. The findings emphasize the need for larger, multicentric investigations with rigorous adjustment for lifestyle and socioeconomic confounders, as well as longitudinal studies that track vitamin D status before and after remission induction in the absence of supplementation. Such work will be critical to determine whether vitamin D deficiency contributes to disease modulation or is a consequence of heightened inflammatory activity. Until then, vitamin D should be regarded as a clinically relevant correlate of RA disease activity, with potential implications for patient monitoring and therapeutic strategies.
